# Mild Cognitive Impairment and Depressive Symptoms in Elderly Patients with Diabetes: Prevalence, Risk Factors, and Comorbidity

**DOI:** 10.1155/2014/179648

**Published:** 2014-11-09

**Authors:** Malgorzata Gorska-Ciebiada, Malgorzata Saryusz-Wolska, Maciej Ciebiada, Jerzy Loba

**Affiliations:** ^1^Department of Internal Medicine and Diabetology, Medical University of Lodz, 251 Pomorska Street, 92-213 Lodz, Poland; ^2^Department of General and Oncological Pneumology, Medical University of Lodz, 22 Kopcinskiego Street, 90-153 Lodz, Poland

## Abstract

The aim of the study was to estimate the prevalence of mild cognitive impairment (MCI), depressive syndrome cases, and its comorbidity, and to identify predictors of these conditions. *Methods*. 276 diabetics elders were screened for MCI and depressive symptoms. Detailed information of history of diabetes, and data of BMI, HbA1c, and blood lipids were collected. *Results*. The prevalence of MCI was 31.5%, depressive syndrome was 29.7%, and MCI with coexisting depressive mood was 9.1%. The logistic regression analysis revealed that variables which increased the likelihood of having been diagnosed with MCI were: higher HbA1c level, previous CVD, hypertension, retinopathy, increased number of comorbidities, and less years of formal education. Significant predictors of having a depressive mood included female gender, single marital status, current and past smoking status, lack of physical activity, higher BMI and total cholesterol level, increased number of comorbidities, history of hypoglycemia, and insulin treatment. Factors associated with both MCI and depressive syndrome were female gender, single marital status, past smoking status, retinopathy, previous CVD or stroke, increased number of comorbidities, and insulin treatment. *Conclusions*. Depressive symptoms, MCI, and its comorbidity are common in elderly subjects with type 2 diabetes. Systematic screening could result in the identification of high-risk patients.

## 1. Introduction

Diabetes mellitus is a common chronic condition worldwide, especially in the elderly population. Older adults with type 2 diabetes (T2DM) are at increased risk for developing micro- and macrovascular complications (e.g., retinopathy, neuropathy, nephropathy, and cardiovascular disease) [1]. Recent studies suggested that T2DM is associated with higher risk of cognitive dysfunction, dementia, and depression in the elderly [2, 3]. The cause of cognitive impairment and depression in type 2 diabetes is unknown, but it is most likely multifactorial. Chronic hyperglycemia, cerebral microvascular disease, severe hypoglycemia, and an increased prevalence of macrovascular disease could play significant role in pathophysiology of brain disturbances. Diabetes is associated with an increased release of inflammatory cytokines, and the excess inflammation may be neurotoxic [4]. Depression, mild cognitive impairment (MCI), and dementia are highly prevalent chronic conditions associated with social, medical, and economic burdens. Although there are several epidemiological studies that have reported the prevalence of mild cognitive impairment (MCI) or depressive syndrome in elderly diabetic population little is known about the comorbidity of these conditions [5, 6]. Data about identification potentially modifiable risk factors for cognitive impairment and depressive syndrome in elderly people with type 2 diabetes are also poor and could be important for future diabetes health care initiatives.

Therefore, the aim of the current study was to (1) estimate the prevalence of MCI, depressive mood, and its comorbidity in the elderly subjects with T2DM and (2) find potential demographic, clinical, and biochemical risk factors associated with cognitive impairment and depressive syndrome.

## 2. Material and Methods

### 2.1. Study Population

A survey was conducted among unselected 276 elders who attended to the outpatient clinic belonging to the Department of Internal Medicine and Diabetology, University Hospital No. 1 in Lodz, Poland. A brief screening for recruitment was conducted by the investigators to identify potential participants. We included patients aged 65 and over with diabetes type 2 diagnosed minimum 1 year earlier, subjects who had been able to understand and cooperate with study procedures. The exclusion criteria were diagnosed depression or dementia, use of possible or known cognition-impairing drugs in the previous 3 months, presence of neoplasm, constant alcohol or substance abuse, severe visual, mobility, or motor coordination impairment, history of head trauma, inflammatory or infectious brain disease, and severe neurological or psychiatric illness.

Written consent was obtained from the participants at the beginning of the study. The first part of visit included a morning blood draw after a 10–12-hour overnight fast, blood pressure measurements, height and weight assessment, and complete physical examination. Then patients had eaten a breakfast followed by capillary glucose level measuring to ensure that participants were not hypoglycemic at the time of cognitive testing. The second part of visit took place in a private area in the clinic. Subjects completed a questionnaire describing baseline demographics and underwent cognitive testing.

### 2.2. Participant Characteristics, Clinical Evaluation, and Risk Factor Assessment

Demographic variables and possible risk factors were recorded in a standardized interview. Weight and height were measured to calculate body mass index (BMI = weight/height^2^ (Kg/m^2^)). The systolic and diastolic blood pressures (mmHg) were measured with the patient in sitting position after 5 minutes of rest. The detailed medical history of diabetes type 2 was taken and includes diabetes duration, currant treatment for diabetes and complications if present, family history of diabetes, comorbid diseases of the patient (hyperlipidemia, hypertension, cardiovascular disease, lung disease, cancer, and gastrointestinal tract diseases), and their treatment. Educational level was recorded in years of education. Smoking was classified as current, past, or never. Physical activity was recorded if any present. Diabetic vascular complications were assessed based on the existence of nephropathy, retinopathy, neuropathy, cardiovascular disease (CVD), and stroke. Hypertension was defined as either a history of hypertension or use of any antihypertensive agents, and hyperlipidemia was defined as use of any lipid lowering agent or an untreated serum LDL cholesterol level 2.6 mmol/L or/and triglycerides 1.7 mmol/L.

### 2.3. Blood Biochemistry

After overnight fasting, blood samples were taken by venipuncture to assess serum levels of glycosylated hemoglobin (HbA1c), total cholesterol, triglycerides, low-density lipoprotein cholesterol (LDL-C), and high-density lipoprotein cholesterol (HDL-C). All the parameters were measured in a centralized laboratory.

### 2.4. Neuropsychological Evaluations

All participants underwent the following tests: the Montreal Cognitive Assessment (MoCA) [7] to evaluate the cognitive impairment, long version of the Geriatric Depression Scale (GDS-30) [8] to assess the depressive mood, Katz Basic Activities of Daily living (BADL), and Lawton Instrumental Activities of Daily Living (IADL) questionnaires to collect information on daily activities [9, 10]. The MoCA tests 8 cognitive domains, visual-spatial ability, attention, executive function, immediate memory, delayed memory, language, abstraction, calculation, and orientation, for a maximum total score of 30. The normal MoCA score is ≥26, with one point added if the subject has fewer than 12 years of formal education. The MoCA is better than other tools to detect MCI in the elderly patients with type 2 diabetes [11]. MCI was diagnosed based on criteria established in the 2006 European Alzheimer's Disease Consortium which are currently available standard test [12, 13]. These criteria included absence of dementia. The cut-off points for MoCA scores (19/30) are recommended for the diagnosis of “dementia” in epidemiological studies. Patients with score 19 and below were excluded from the study as dementia and sent to psychiatrist for further care. The criteria mentioned above included also absence of major repercussions on daily life (in our study, measured by Katz BADL and Lawton IADL).

This interview was followed by Geriatric Depression Scale (GDS) for mood assessment [8]. GDS consists of 30 items. Scores range from 0 to 9 was considered as normal and from 10 to 19 was considered to have depressive symptoms. Patients with score 20 and above were excluded from the study as severe depressive symptomatology and sent to psychiatrist for further diagnosis.

According to criteria mentioned above 276 older subjects with diabetes type 2 were selected into groups: patients with MCI, patients without MCI, patients with depressive mood, patients without depressive mood, patients with MCI and depressive mood, and patients without MCI and depressive mood.

### 2.5. Ethics

The study was operated in accordance with the World Medical Association's Declaration of Helsinki. Each participant was assigned a number by which he/she was identified to keep his or her privacy. The approval was obtained from the independent local Ethics Committee of Medical University of Lodz.

## 3. Statistical Analysis

The descriptive statistics for the categorical variables were tested using the Chi-square and the continuous variables using the Student's *t*-test or the Mann-Whitney *U* tests whenever applicable. Three multivariate logistic regression models were generated to identify the influential factors associated with the presence of MCI, depressive syndrome, and both. The dependent factors were set as having MCI or having depressive mood or having both MCI and depressive mood. The univariate analysis included the variables that were found to be significant in the models. The independent variables entered in the model at step one were gender, age, education, marital status, smoking status, physical activity, duration of diabetes, the body mass index, HbA1c and lipids levels, treatment of DM 2, micro- and macrovascular complications, previous HA or use of HA drugs, hyperlipidemia, number of comorbid conditions, and presence of hypoglycemia. Odds ratios (OR) were computed and presented with the 95% interval of confidence (CI). The nonsignificant predictors (*P* > 0.05) were subsequently removed on a backward stepwise basis. A *P* value of less than 0.05 was considered statistically significant. Statistica 10.0 (StatSoft, Poland, Krakow) was used for analysis.

## 4. Results

The demographic and clinical characteristics of the study group have been presented in Table 1. The prevalence of MCI in elderly patients with type 2 diabetes was 31.5% (n-87), and the prevalence of subjects with depressive syndrome was 29.7% (n-82). We also evaluated 25 participants (9.1%) with both MCI and depressive mood. Compared with the group without MCI, patients with MCI were significantly older and less educated and had a longer duration of diabetes; more were diagnosed with cardiovascular disease, hypertension, hyperlipidaemia, retinopathy, nephropathy, and other comorbidities and more had a history of hypoglycemia. The level of HbA1c and triglycerides was higher and HDL was lower in MCI group. Patients with depressive syndrome were more likely to be female, single, and current and past smokers and had less physical activity, higher BMI, and a longer duration of diabetes; more were diagnosed with neuropathy and other comorbidities, had a history of hypoglycemia, and were treated with insulin. The level of total cholesterol and LDL cholesterol was higher in group with depressive mood. The characterization of 25 subjects with both MCI and depressive syndrome is presented in Table 1.

The univariate logistic regression models revealed many factors which are associated with the diagnosis of MCI, depressive syndrome, and both (Table 2). Finally we constructed three multivariate logistic regression models to determine the predictors of MCI, depressive status, and both (Table 3). The first model showed that variables which increased the likelihood of having been diagnosed with MCI were higher level of HbA1c, presence of previous CVD, hypertension, retinopathy, increased number of comorbidities, and less years of formal education. Significant predictors of having a depressive mood included female gender, single marital status, current and past smoking status, lack of physical activity, higher BMI and total cholesterol level, increased number of comorbidities, presence of history of hypoglycemia, and insulin treatment. Factors associated both with MCI and depressive syndrome were as follows: female gender, single marital status, past smoking status, retinopathy, presence of previous CVD or stroke, increased number of comorbidities, and insulin treatment.

## 5. Discussion

Type 2 diabetes is a complex metabolic disease, caused by reduced insulin sensitivity and relative insulin deficiency. Coexisting disorders, including hypertension, dyslipidaemia, and obesity, contribute to the severity of type 2 diabetes. One of target end-organ in type 2 diabetes is the brain. The cause of diabetes-related brain dysfunction is difficult to establish because of the prevalence of several comorbidities, each of which might affect it. There are many studies which had investigating prevalence of cognitive function and depression disorder in type 2 diabetes. However none of them had described the coexistence of MCI and depressive symptoms in diabetic subjects and factors associated with the presence of both these conditions. The prevalence of MCI in our study was 31.5% and it was comparable to other data in the worldwide literature. One population-based study reported that the incidence of MCI in diabetic subjects was around 28% [2]. A meta-analysis of longitudinal studies showed that diabetes increased the risk of mild cognitive impairment by 21% [14]. Data from large population studies suggest that chronic hyperglycaemia is a risk for cognitive impairment in diabetes type 1 and type 2 [15, 16]. In agreement with many other results our study reported that higher level of HbA1c was a risk factor for diagnosis of MCI. The pathomechanism underlying this process is complex and can include increased formation advanced glycation end products (AGEs), oxidative stress and inflammation, accumulation of sorbitol, and development of a hyperosmotic state in nerve cells that leads to edema and impaired brain function, impaired insulin homeostasis in the brain, and insulin resistance syndrome [4]. Chronic hyperglycaemia and long duration of diabetes are both associated with increased development of cognitive dysfunction, as is the presence of vascular risk factors (e.g., hypertension, hypercholesterolaemia, and obesity) and microvascular and macrovascular complications [17]. The Edinburgh type 2 diabetes study showed that general cognitive ability was significantly lower in people with moderate-to-severe diabetic retinopathy than in those without retinopathy [18]. In our study presence of retinopathy increased a likelihood of having MCI or both MCI and depressive mood.

The underlying mechanism of the association between hypertension and cognitive impairment in diabetics might include cerebrovascular diseases and vascular dysfunction. Patients with diabetes have an increased risk in thrombotic stroke, and vascular disease has long been hypothesized to contribute to abnormalities in cognition in such patients [4, 19]. The hypertension can cause vascular occlusion and compromise cerebral infarction, alter the structure of cerebral blood vessels, and change blood supply to the brain [20, 21]. We had also noticed that patients with MCI had higher level of triglyceride and lower level of HDL cholesterol. The hyperlipidemia was significant predictor of diagnosis with MCI. In agreement with our results, other studies had showed similar association and explained that hyperlipidemia itself causes denaturation of neurons responsible for cognitive function or accelerates the progression of atherosclerosis, which leads to MCI [22, 23]. Finally the education level is well-known factor strongly associated with cognitive function. Previous studies have shown that diabetic patients with lower levels of education have more long-term complications, more cardiovascular diseases, and a greater risk of mortality [24].

In our study we noticed also a high prevalence of depressive symptoms (29.7%) among elderly patients with type 2 diabetes. Since depression can cause cognitive deficits, patients with diagnosed depression were excluded. Other studies performed in elderly general population (but not only diabetics) had showed also high prevalence [6, 25]. In the Cardiovascular Health Study Cognition Study approximately 41% of participants had depressive mood [25]. In the large population study performed among Japanese elders the prevalence of depressive symptoms was 11.5% [6]. The authors had found high coexistence rate for depression and MCI and concluded that individuals with MCI were more likely to develop depressive symptoms and vice versa. In agreement with many other results our study revealed significant predictors of having a depressive mood: female gender, single marital status, current and past smoking status, lack of physical activity, higher BMI, and total cholesterol level [26, 27]. Some researchers also indicated that depression is highly prevalent among diabetics and the risk of depression might be increased in the presence of other comorbid conditions [27]. We found that the strongest predictors of being diagnosed with MCI and depressive symptoms were micro- and macrocomplications of diabetes like presence of retinopathy, previous cardiovascular disease, and stroke. Other data had also showed that diabetic complications and mortality were greater among depressed patients. The microvascular and macrovascular complications of diabetes are augmented by the presence of depression in diabetes thus contributing to the increased mortality rate in this population [28].

The mechanism underlying the linkage between MCI and depression has to be elucidated. It have been hypothesized that small vessel diseases in the brain (white matter lesions and lacunae) affect cognitive function in older diabetics without overt dementia or symptomatic stroke [29]. The contribution of small vessel disease to depression has been also described. In contrast to “vascular disease” pathogenesis other researchers had found that depressive symptoms were associated with increased risk of MCI, and this association was independent of underlying vascular disease [25]. The authors had explained that depressive symptoms in the absence of overt cognitive impairment may reflect the early signs of a neurodegenerative disease or that depression leads to damage in the hippocampus through a glucocorticoid cascade.

## 6. Conclusions

Depressive symptoms and MCI are common in elderly subjects with type 2 diabetes. There is also coexistence of these conditions in some patients. Although there have been many significant demographic, clinical, and biochemical risk factors associated with cognitive impairment and depressive syndrome the causative mechanisms of diabetes on brain's complication are still unclear. Systematic screening and early intervention strategies could result in the identification of high-risk patients and the improvement of the effectiveness of the treatment.

## Figures and Tables

**Table 1:** Demographic and clinical characteristics of type 2 diabetic elderly patients.

	All subjects	Type 2 diabetes with MCI	Type 2 diabetes without MCI	Type 2 diabetes with depressive syndrome	Type 2 diabetes without depressive syndrome	Type 2 diabetes with MCI and depressive syndrome	Type 2 diabetes without MCI and depressive syndrome
Number of patients	276	87 (31.5%)	189 (68.5%)	82 (29.7%)	194 (70.3%)	25 (9.1%)	251 (90.9%)
Male/female	127/149	34/53	93/96	15/67	112/82^#^	4/21	123/128^$^
Age (years)	73.6 ± 4.8	75.7 ± 4.6	72.6 ± 4.6^*^	74.4 ± 5.0	73.2 ± 4.7	78.0 ± 5.3	73.1 ± 4.5^$^
Education years	11.3 ± 2.4	9.7 ± 1.8	12.0 ± 2.2^*^	11.2 ± 2.4	11.3 ± 2.3	9.5 ± 1.7	11.5 ± 2.3^$^
Marital status: single/married	127/149 (46.01%)	45/42	82/107	58/24	69/125^#^	21/4	106/145^$^
Current smoking	19 (6.88%)	6 (6.89%)	13 (6.87%)	13 (15.8%)	6 (3.09%)^#^	4 (16%)	15 (5.97%)
Had ever smoked	93 (33.7%)	26 (29.8%)	67 (35.4%)	47 (57.3%)	46 (23.7%)^#^	14 (56%)	79 (31.4%)^∧^
Lack of physical activity (%)	105 (38.04%)	26 (29.88%)	79 (41.79%)	57 (69.51%)	48 (24.74%)^#^	14 (56%)	91 (36.25%)
Duration of DM2 (years)	8.69 ± 6.23	11.25 ± 6.29	7.51 ± 5.85^*^	10.83 ± 6.72	7.78 ± 5.8^#^	12.8 ± 6.23	8.28 ± 6.09^∧^
BMI (Kg/m^2^)	29.9 ± 3.67	30.4 ± 3.59	29.6 ± 3.68	32.03 ± 3.6	29.02 ± 3.32^#^	31.8 ± 3.2	29.7 ± 3.6^$^
HbA1c (%)	7.24 ± 0.68	7.73 ± 0.71	7.01 ± 0.54^*^	7.35 ± 0.77	7.19 ± 0.64	8.02 ± 0.68	7.16 ± 0.63^$^
CHOL (mmol/L)	10.3 ± 2.18	10.31 ± 2.2	10.29 ± 1.71	11.59 ± 2.19	9.75 ± 1.93^#^	10.75 ± 2.1	10.25 ± 2.18
LDL (mmol/L)	6.06 ± 1.67	6.01 ± 1.64	6.08 ± 1.73	6.93 ± 1.83	5.69 ± 1.5^#^	6.54 ± 1.89	6.01 ± 1.68
TG (mmol/L)	9.65 ± 2.23	10.59 ± 2.68	9.22 ± 1.84^*^	9.82 ± 2.4	9.58 ± 2.16	11.33 ± 2.63	9.48 ± 2.12^$^
HDL (mmol/L)	2.5 ± 0.51	2.3 ± 0.6	2.67 ± 0.42^*^	2.52 ± 0.47	2.57 ± 0.53	2.15 ± 0.45	2.59 ± 0.5^$^
Treatment							
Insulin	130 (47.1%)	42 (48.2%)	88 (46.5%)	60 (73.2%)	70 (36.0%)^#^	19 (76%)	111 (44.2%)^∧^
OAD	222 (80.4%)	71 (81.6%)	151 (79.8%)	66 (80.4%)	157 (80.9%)	20 (80%)	212 (84.4%)
Complications							
Previous CVD	109 (39.5%)	71 (81.6%)	38 (20.1%)^*^	33 (40.2%)	76 (39.17%)	23 (92%)	86 (34.2%)^$^
Stroke	14 (5.07%)	7 (8.04%)	7 (3.7%)	7 (8.53%)	7 (3.6%)	5 (20%)	9 (3.58%)
Previous HA or use of HA drugs	213 (77.17%)	80 (91.95%)	138 (73.01%)^*^	60 (73.17%)	153 (78.86%)	21 (84%)	192 (76.49%)
Hyiperlipidemia	218 (78.9%)	81 (93.1%)	132 (69.8%)^*^	76 (92.6%)	142 (73.2%)^#^	24 (96%)	194 (77.3%)^∧^
Microvascular complication							
Retinopathy	121 (43.8%)	61 (70.1%)	60 (31.7%)^*^	35 (42.6%)	86 (44.32%)	18 (72%)	103 (41.0%)^$^
Nephropathy	97 (35.1%)	43 (49.4%)	54 (28.5%)^*^	27 (32.9%)	70 (36.08%)	11 (44%)	86 (34.26%)
Neuropathy	56 (20.2%)	20 (22.9%)	36 (19.04%)	33 (40.2%)	23 (11.8%)^#^	10 (40%)	46 (18.4%)^∧^
Co-morbidity (*n*)	4.66 ± 3.11	7.07 ± 3.22	3.55 ± 2.33 ^*^	6.43 ± 3.07	3.91 ± 2.81^#^	9 ± 2.82	4.22 ± 2.79^$^
Hypoglycemia	117 (42.3%)	60 (68.9%)	57 (30.1%)^*^	60 (73.1%)	57 (29.3%)^#^	22 (88%)	95 (37.8%)^$^
MoCA score	25.6 ± 3.07	21.6 ± 1.5	27.4 ± 1.3^*^	26.01 ± 3.2	25.5 ± 2.9	21.8 ± 1.7	25.9 ± 2.9^$^
GDS-30 score	6.8 ± 6.5	7.2 ± 6.2	6.7 ± 6.6	15.9 ± 2.8	3 ± 2.7^#^	16 ± 2.7	5.9 ± 6.07^$^
Katz BADL score	4.96 ± 0.2	5 ± 0.2	4.96 ± 0.2	4.96 ± 0.1	5 ± 0.2	4.96 ± 0.2	4.96 ± 0.2
Lawton IADL score	7.9 ± 0.1	8 ± 0.1	7.9 ± 0.07	7.9 ± 0.1	8 ± 0.07	7.96 ± 0.2	7.9 ± 0.08

^*^ *P* < 0.001, comparing participants with MCI and those without MCI, ^#^ *P* < 0.001, comparing participants with depressive syndrome and without depressive syndrome, and ^$^ *P* < 0.001, ^∧^ *P* < 0.02, comparing participants with MCI and depressive syndrome and without MCI and depressive syndrome.

DM2: diabetes type 2, OAD: oral antidiabetic drug, CVD: cardiovascular disease, HA: hypertension, BMI: body mass index, MoCA: Montreal Cognitive Assessment, GDS: Geriatric Depression Scale, BADL: Basic Activities of Daily living, and IADL: Instrumental Activities of Daily Living.

The Student *t*-test, Mann-Whitney *U* test, or Chi-square test was used to test for significant differences.

**Table 2:** Factors associated with MCI, depressive syndrome, and both (univariate logistic regression model).

Associated parameters with OR (95% CI)	MCI	Depressive syndrome	Both (MCI and depressive syndrome)
Gender: female	—	2.47 (1.8–3.38)	2.25 (1.29–3.88)
Age (years)	1.15 (1.08–1.22)	—	1.24 (1.12–1.36)^*^
Education (years)	0.53 (0.44–0.63)	—	0.65 (0.49–0.78)^*^
Marital status: single	—	2.09 (1.58–2.76)	2.68 (1.55–4.64)^*^
Current smoking	—	2.43 (1.46–4.02)	—
Had ever smoked	—	2.08 (1.58–2.74)	1.67 (1.09–2.52)
Lack of physical activity	—	2.63 (1.98–3.5)	—
Duration of DM2 (years)	1.1 (1.05–1.15)	1.08 (1.03–1.16)	1.086 (1.03–1.14)
BMI (Kg/m^2^)	—	1.02 (1.01–1.04)	1.16 (1.05–1.3)
HbA1c (%)	5.47 (3.45–8.67)	—	5.97 (3.02–11.84)^*^
CHOL (mmol/L)	—	1.02 (1.01–1.04)	—
LDL (mmol/L)	—	1.02 (1.01–1.03)	—
TG (mmol/L)	1.02 (1.01–1.03)	—	1.02 (1.01–1.03)^*^
HDL (mmol/L)	0.9 (0.87–0.94)	—	0.88 (0.83–0.94)^*^
Insulin	—	2.19 (1.65–2.92)	1.998 (1.242–3.26)
Previous CVD	4.19 (3.04–5.8)	—	4.69 (2.25–9.78)^*^
Stroke	—	—	2.59 (1.43–4.68)
Previous HA or use of HA drugs	2.41 (1.55–3.76)	—	—
Hyperlipidemia	2.06 (1.35–3.12)	2.15 (1.38–3.36)	—
Retinopathy	2.25 (1.7–2.96)	—	1.92 (1.22–3.03)
Nephropathy	1.56 (1.2–2.03)	—	—
Neuropathy	—	2.23 (1.64–3.05)	1.72 (1.12–2.65)
Comorbidity (*n*)	1.53 (1.37–1.71)	1.31 (1.19–1.43)	1.63 (1.38–1.93)^*^
Hypoglycemia (%)	2.27 (1.72–2.98)	2.56 (1.92–3.42)	3.47 (1.87–6.43)^*^

^*^ *P* < 0.001 and others *P* < 0.05.

**Table 3:** Factors associated with MCI, depressive syndrome, and both (multivariate logistic regression model).

	*P* value	OR (95% CI)
*Associated parameters with MCI *		
HbA1c	0.001	2.98 (1.56–5.68)
Previous HA or use of HA drugs	0.003	2.77 (1.39–5.48)
Retinopathy	0.021	1.63 (1.07–2.45)
Previous CVD	0.000	2.9 (1.91–4.04)
Comorbidity (*n*)	0.005	1.24 (1.06–1.43)
Years of education	0.000	0.65 (0.52–0.82)

*Associated parameters with depressive syndrome *		
Gender: female	0.001	2.43 (1.47–4.01)
Marital status: single	0.004	1.93 (1.23–3.04)
Current smoking	0.003	3.74 (1.56–8.91)
Had ever smoked	0.042	1.63 (1.02–2.61)
Lack of physical activity	0.000	3.1 (1.92–5.02)
BMI (Kg/m^2^)	0.002	1.2 (1.06–1.36)
CHOL (mmol/L)	0.015	1.02 (1.0–1.03)
Insulin	0.003	2.01 (1.26–3.2)
Comorbidity (*n*)	0.035	1.17 (1.01–1.36)
Hypoglycaemia	0.016	1.85 (1.12–3.04)

*Associated parameters with MCI and depressive syndrome *		
Gender: female	0.045	2.14 (1.06–4.33)
Marital status: single	0.005	2.93 (1.37–6.25)
Had ever smoked	0.027	2.16 (1.09–4.25)
Retinopathy	0.034	2.14 (1.06–4.33)
Previous CVD	0.002	3.97 (1.65–9.57)
Stroke	0.025	3.41 (1.16–9.9)
Insulin	0.02	2.47 (1.15–5.28)
Comorbidity (*n*)	0.000	1.56 (1.23–1.98)
